# Potential planting regions of *Pterocarpus santalinus* (Fabaceae) under current and future climate in China based on MaxEnt modeling

**DOI:** 10.1002/ece3.11409

**Published:** 2024-05-30

**Authors:** Xiao‐Feng Zhang, Mir Muhammad Nizamani, Chao Jiang, Fa‐Zhi Fang, Kun‐Kun Zhao

**Affiliations:** ^1^ Hainan Academy of Forestry (Hainan Academy of Mangrove) Haikou China; ^2^ Department of Plant Pathology, College of Agriculture Guizhou University Guiyang China; ^3^ Jinxian County No. 3 Middle School Nanchang China; ^4^ Tropical Crops Genetic Resources Institute Chinese Academy of Tropical Agricultural Sciences Haikou China

**Keywords:** China, climate change, MaxEnt, planting suitability regionalization, *Pterocarpus santalinus*

## Abstract

This study modeled the habitat distribution of *Pterocarpus santalinus*, a valuable rosewood species, across China under current and future climate scenarios (SSPs126, SSPs245, and SSPs585) using MaxEnt. Our findings reveal that the current suitable habitat, spanning approximately 409,600 km^2^, is primarily located in the central and southern parts of Guangdong, Guangxi, Fujian, and Yunnan, as well as in the Hainan provinces, along with the coastal regions of Taiwan, and the Sichuan–Chongqing border. The habitat's distribution is significantly influenced by climatic factors such as temperature seasonality (bio4), mean temperature of the wettest quarter (bio8), annual mean temperature (bio1), and annual precipitation (bio12), while terrain and soil factors play a lesser role. Under future climate scenarios, the suitable habitat for *P. santalinus* is projected to expand, with a northeastward shift in its distribution center. This research not only sheds light on the geoecological characteristics and geographical distribution of *P. santalinus* in China but also offers a scientific basis for planning its cultivation areas and enhancing cultivation efficiency under changing climate conditions.

## Graphical abstract

Potential planting regions of *Pterocarpus santalinus* under current and future climate in China based on MaxEnt model. This study is vital for developing adaptive strategies for the cultivation and conservation of this economically significant species in the face of ongoing climatic shifts.

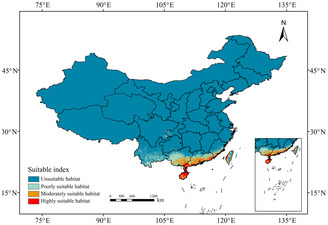

## 1 INTRODUCTION

The suitable habitat of tree species is influenced by a combination of factors, including biological characteristics and interactions with both biological and environmental factors (Chen et al., 2010; Urban et al., 2013). Global climate change, particularly alterations in temperature and precipitation, directly impacts species distribution patterns and growth areas (Mantyka‐Pringle et al., 2012). Evidence indicates a general rise in global temperatures due to greenhouse gas emissions, projected to cause a 0.3–4.8°C increase in global average temperature by the century's end (Masson‐Delmotte et al., 2021; Stocker et al., 2013). This warming triggers extreme weather events and biotic stress, posing new adaptability challenges for species and altering spatial distribution patterns (Hughes, 2000; Niu et al., 2014).

However, there exists a significant gap in the literature regarding the habitat range fluctuation of tree species due to these evolving climatic conditions, particularly in diverse ecological zones. In the eastern United States, 58.7% of tree species' population ranges are shrinking, with 20.7% migrating northward due to climate change (Zhu et al., 2012). There is a decrease in the high suitability habitat of Dalbergia cultrata with warming climates (Liu et al., 2019). Similarly, there is an expansion of suitable planting areas for coffee in Yunnan, China, toward higher altitudes and latitudes under future climate scenarios (Zhang et al., 2021). These studies underscore the profound and irreversible impact of climate change on terrestrial vegetation (Gang et al., 2017), highlighting the urgent need for comprehensive research into species' adaptations and responses to climate change (Zhao et al., 2020).

Species distribution models (SDMs) are pivotal in evaluating habitat suitability across different regions by integrating species distribution data with environmental factors (Li et al., 2020; Yi et al., 2018). Among these models, the MaxEnt model, based on the principle of maximum entropy, is a classical approach for simulating the geographic distribution of species (Xu et al., 2014). MaxEnt stands out for its efficiency, ability to analyze both continuous and classified environmental variables, and high accuracy even with limited sample sizes (Hu et al., 2015; Li et al., 2019; Wang et al., 2010). The research indicated that predictions from the MaxEnt model were highly consistent with the known distribution areas of the species, and it demonstrated greater accuracy in comparison to other models (Babar et al., 2012; Jinga & Ashley, 2019). Consequently, this model has been applied across various domains, including the protection of endangered plant (Gao et al., 2022; Yi et al., 2016), scientific planting strategies (Xia et al., 2023; Zhang et al., 2021), species richness estimates (Nizamani et al., 2023), management of invasive alien species (Yan et al., 2020), natural disaster assessment (Javidan et al., 2021), and pest prevention and control (Kumar et al., 2015).

*Pterocarpus santalinus*, also known as Red Sanders, is a deciduous tree from the Fabaceae family. This species has specific requirements for its growth environment, such as sunlight, moisture, soil, and altitude. It is endemic to the southern parts of the Eastern Ghats, India, and is primarily found in the forest tracts of Chittoor and Kadapa districts. Historically, *P. santalinus* is renowned for its medicinal and timber value. Research indicates that *P. santalinus* contains valuable phytochemicals like santalin, triterpenes, and flavonoids, which contribute to its anti‐inflammatory, anti‐cancer, and hepatoprotective properties (Navada & Vittal, 2014). Beyond its medicinal properties, *P. santalinus* is also prized for extracting natural dyes and crafting high‐end wooden products. However, the high demand and economic value of *P. santalinus* have led to severe depletion of its wild resources (Zhang et al., 2019). In consideration of species conservation, the MaxEnt model has been used to delineate potential protected areas for *P. santalinus* in its native habitat (Babar et al., 2012). Similarly, other species within the Pterocarpus face analogous challenges, such as *P. marsupium* (Khanal et al., 2022) and *P. erinaceus* (Adjonou et al., 2020; Biaou et al., 2023). In order to resolve the demand for *P. santalinus* in Chinese market, rosewood plantations have emerged as a viable solution, offering economic benefits and employment opportunities in regions like Hainan, Guangdong, Guangxi, Yunnan, and Fujian in China (Chen & Zeng, 2018; Krainovic et al., 2018). Despite the expansion of cultivation, *P. santalinus* is sensitive to hydrothermal conditions, and indiscriminate planting can lead to numerous problems (Xu, 2011). Given the uncertainty about suitable planting areas for *P. santalinus* in China and the potential impact of climate change on the future suitability of these areas, the need for accurate predictions is urgent.

In this study, the suitable habitat of *P. santalinus* under current and future climate conditions was evaluated by the MaxEnt model based on the information collected from the specimen record and field investigation. The objectives of this study include: (1) identify the suitable planting areas for rosewood tree species under the current climate condition; (2) identify the key environmental factors that affect the growth of rosewood tree species; (3) predict the changes in suitable planting areas for rosewood tree species under future climate conditions. The solution to these problems can provide a scientific basis for the planting layout and efficient cultivation of *P. santalinus* in China.

## 2 MATERIALS AND METHODS

### 2.1 Data collection

#### 2.1.1 Species occurrence locations

**FIGURE 1:**
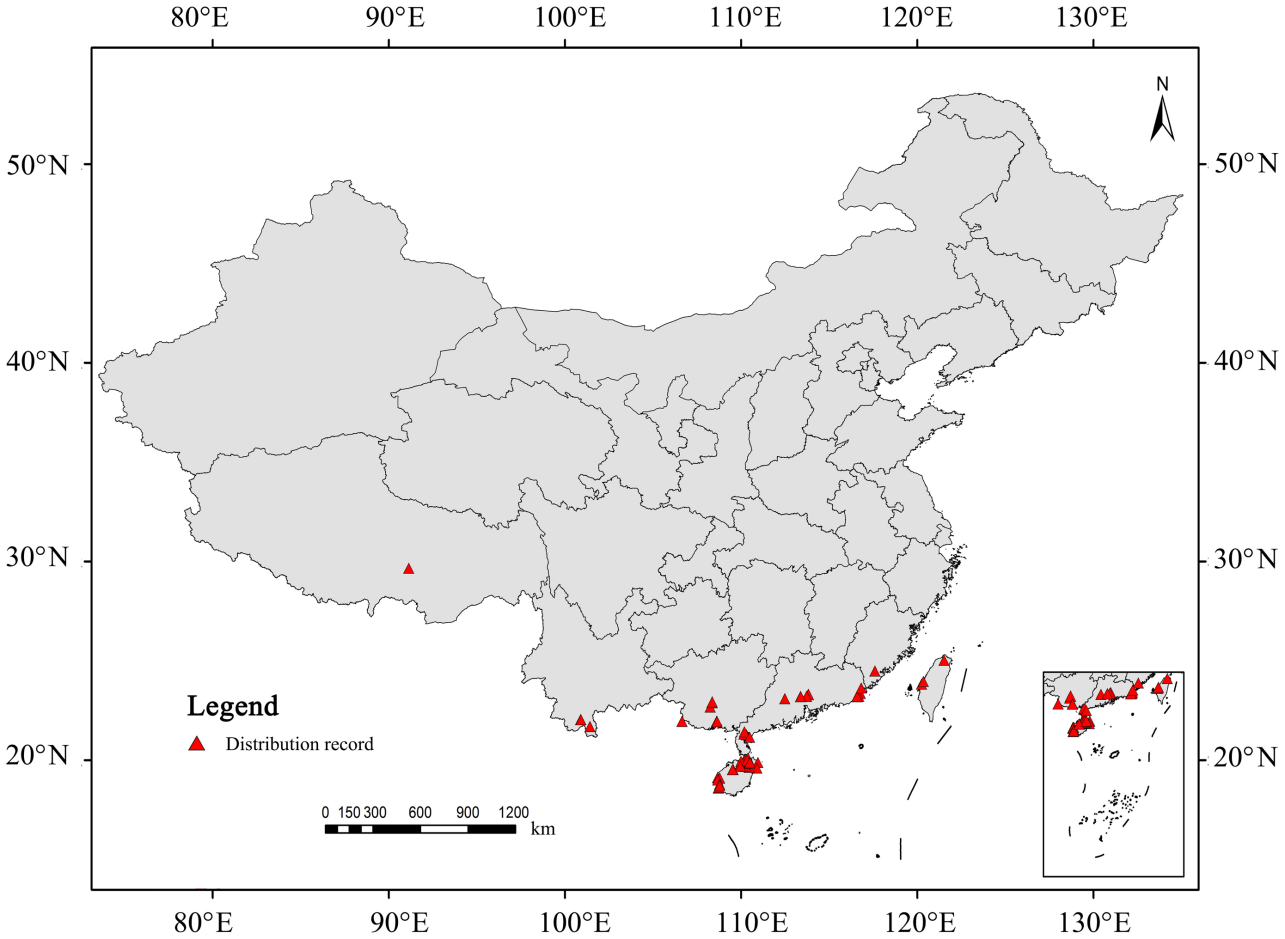
Spatial distribution of occurrence records of *Pterocarpus santalinus* in China.

In this study, we identified the occurrence locations of *P. santalinus* using a three‐pronged approach: (1) a comprehensive review of relevant literature; (2) extensive field investigations; and (3) data acquisition from online databases, specifically the Global Biodiversity Information Facility (GBIF, https://www.gbif.org) and the National Specimen Information Infrastructure (NSII, http://www.nsii.org.cn/2017/home.php). To ensure accuracy, we meticulously filtered out duplicate records and entries lacking precise location details. Additionally, to mitigate the impact of spatial autocorrelation and the consequent overfitting, we used ArcGIS to ensure that the distance between each occurrence location exceeded 10 km. This rigorous process culminated in the identification of 62 distinct locations of *P. santalinus*, as illustrated in Figure 1.

#### 2.1.2 Environmental parameters

In our study, we sourced a comprehensive set of environmental variables relevant to the distribution of *P. santalinus* from the WorldClim database (https://www.worldclim.org/data/index.html; Hijmans et al., 2005). This dataset included 19 bioclimatic variables (bio1–bio19) with a spatial resolution of 30 s, along with three topographic factors: altitude, slope, and aspect. Additionally, global soil data were obtained from the Harmonized World Soil Database v 1.2 (HWSD; http://www.fao.org/soils‐portal/soil‐survey/soil‐maps‐and‐databases/harmonized‐world‐soil‐database‐v12/en/), and six soil parameters were extracted using ArcGIS 10.2. These parameters included soil bulk density (t_bden), soil pH (t_ph), sand content (t_sand), clay content (t_clay), organic carbon content (t_oc), and gravel percentage (t_gravel). All these environmental variables were converted into ASCII format using ArcGIS 10.2.

Recognizing the potential bias in prediction results due to the correlation between environmental variables (Hu & Liu, 2014), we undertook a correlation analysis of the 19 bioclimatic factors using ENMTools (Warren et al., 2010). To optimize the model's predictive accuracy and reduce redundancy (Ashcroft et al., 2011; Yi et al., 2016), variables with a correlation coefficient less than 0.8 were retained, while those with higher correlations had the less ecologically significant ones removed, as depicted in Figure S1 (Liu et al., 2019). This process led to the selection of 17 variables out of the initial 28 for our predictive analysis (Table 1). For future climate modeling, we chose the BCC‐CSM2‐MR dataset (https://www.worldclim.org/data/cmip6/cmip6climate.html) to project scenarios for the 2050s (2041–2060), 2070s (2061–2080), and 2090s (2081–2100) with a spatial resolution of 30 s (Zhou et al., 2020). Three Shared Socio‐economic Pathways (SSPs)—SSPs126 (lowest greenhouse gas emission scenario), SSPs245 (moderate greenhouse gas emission scenario), and SSPs585 (highest greenhouse gas emission scenario)—were selected to provide a more scientific depiction of future climate change (Yang et al., 2023).

**TABLE 1:** The 17 environmental variables used for model prediction.

Index	Abbreviation	Description
Climatic variables	bio1	Annual mean temperature
	bio2	Mean diurnal range (mean of monthly (max temp − min temp))
	bio3	Isothermality (BIO2/BIO7) (×100)
	bio4	Temperature seasonality (standard deviation ×100)
	bio8	Mean temperature of wettest quarter
	bio12	Annual precipitation
	bio14	Precipitation of driest month
	bio15	Precipitation seasonality (coefficient of variation)
Terrain variables	alt	Altitude
	slo	Slope
	asp	Aspect
Soil variables	t_bden	Soil bulk density
	t_ph	Soil pH
	t_sand	Sand content
	t_clay	Clay content
	t_oc	Organic carbon content
	t_gravel	Gravel percentage

### 2.2 Planting suitability regionalization

We conducted the planting suitability regionalization for *P. santalinus* in China using the MaxEnt software (version 3.3.3; Phillips et al., 2006). In our approach, we allocated 75% of the distribution points as training data and the remaining 25% as test data for model validation, adhering to the methodology suggested by Moreno et al. (2011). The model utilized both environmental factors and the distribution data of *P. santalinus* for prediction purposes. We set the algorithm to run for 500 iterations or until the convergence threshold reached a minimal value of 0.00001, whichever occurred first. To ensure robustness, this process was repeated 50 times.

The accuracy of the model's predictions was evaluated using the Receiver Operating Characteristic (ROC) curve and the Area Under the Receiver Operating Characteristic Curve (AUC). The AUC provides a reliable measure of the model's performance, with higher values indicating greater predictive accuracy. Additionally, the contribution rate of each environmental variable was analyzed to identify the primary factors influencing the species' distribution. This was complemented by a Jackknife test, which further ascertained the importance of individual environmental variables in the model.

Based on the method described by Zhao et al. (2021), we classified the potential habitat of *P. santalinus* into four categories: high suitability (0.5–1.0), moderate suitability (0.3–0.5), low suitability (0.1–0.3), and not suitable (0–0.1). This categorization provides a nuanced understanding of the varying degrees of habitat suitability across different regions in China. To forecast the migration trajectory of the centroid of suitable habitats for *P. santalinus* under future climate change, we employed ArcGIS software to analyze the shifting locations based on the methodology described by Yue et al. (2011).

## 3 RESULTS

### 3.1 Suitable habitat for *P. santalinus* in the current climate and model accuracy

**FIGURE 2:**
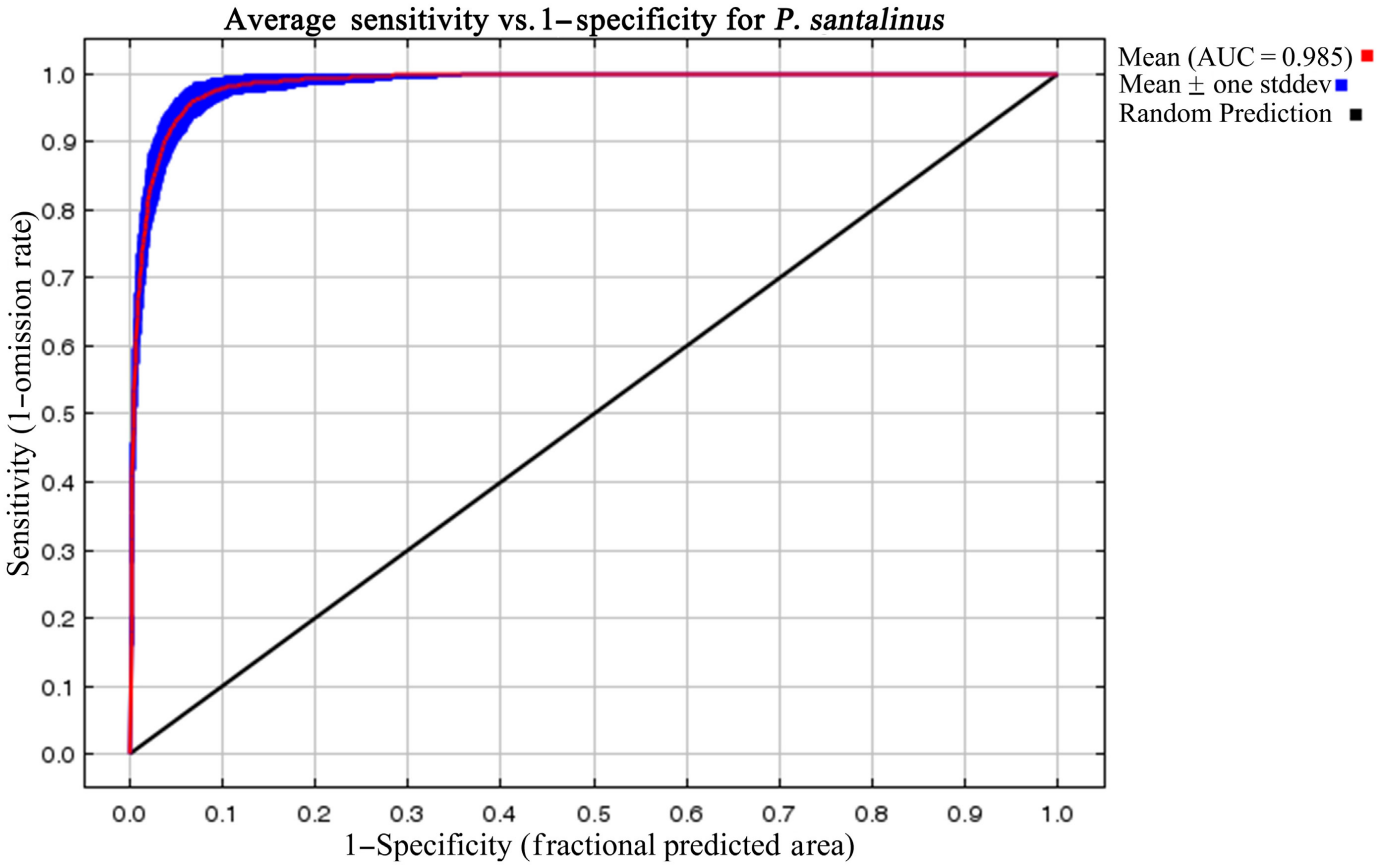
Receiver operating characteristic (ROC) curve of *Pterocarpus santalinus* under the current climate.

**FIGURE 3:**
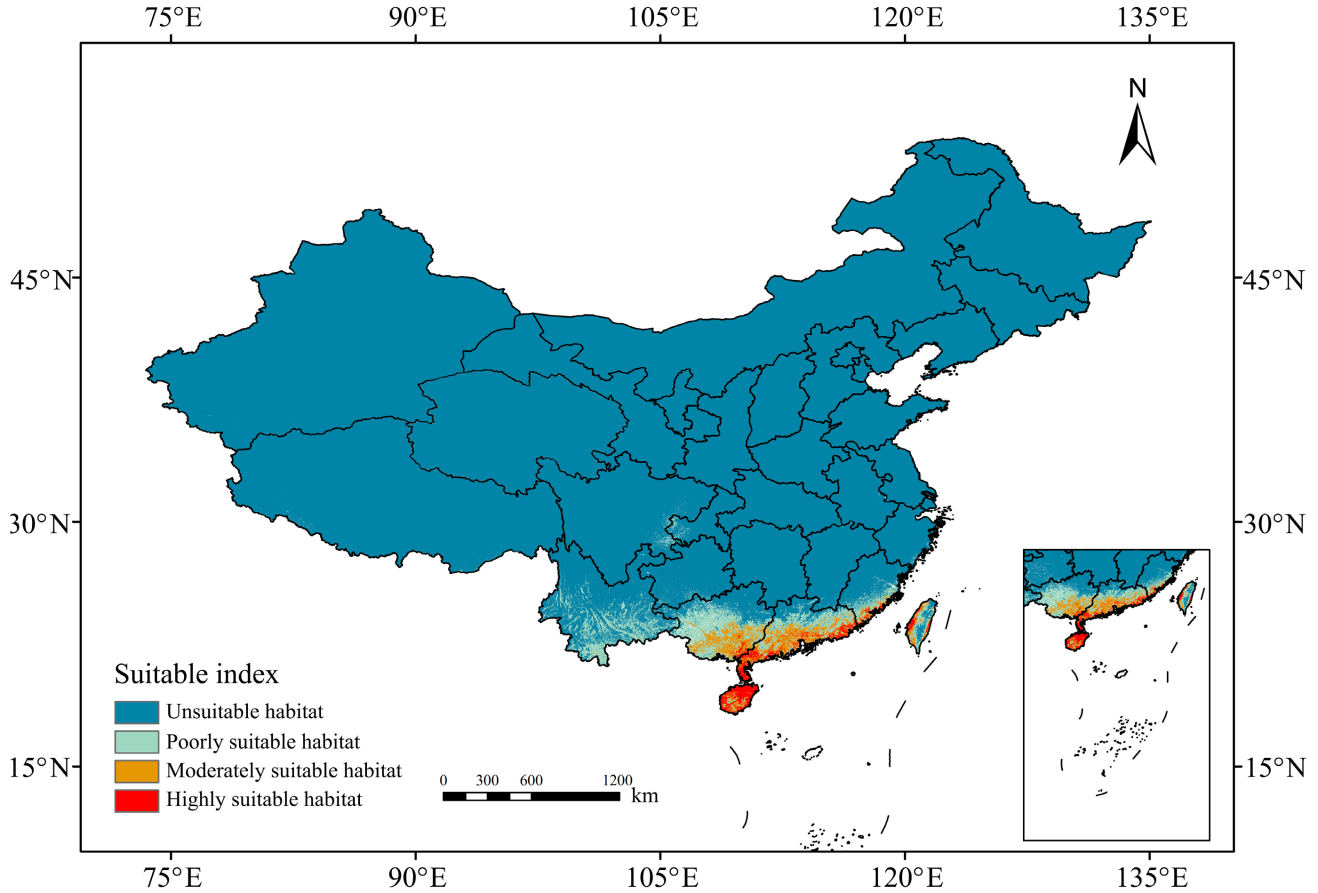
Suitable habitat for *Pterocarpus santalinus* in China under the current climate.

The MaxEnt model's predictive accuracy for *P. santalinus* distribution was exceptionally high, as indicated by an average training area under the curve (AUC) of 0.985 ± 0.003 across replicate runs (Figure 2). This level of accuracy underscores the model's reliability in forecasting the species' distribution. According to our predictions, the current climatic range for *P. santalinus* in China spans from 97°31′ E to 121°48′ E in longitude and 18°10′ N to 30°39′ N in latitude. The species is primarily found in the central and southern parts of Guangdong, Guangxi, Fujian, and Yunnan, as well as in Hainan provinces, along with the coastal areas of Taiwan, and near the border between Sichuan and Chongqing (Figure 3).

The total suitable habitat for *P. santalinus* in these regions covers approximately 409,600 km^2^, which constitutes about 4.26% of China's national area. Notably, the majority of this habitat, accounting for 60.57%, falls into the category of low suitability. These areas are mainly located in the central and southern parts of Guangdong, Guangxi, Fujian, and Yunnan, the central Taiwan, and the border regions between Sichuan and Chongqing. On the other hand, the moderately and highly suitable areas, which make up 39.43% of the *P. santalinus* habitat, are predominantly concentrated along the coasts of Guangdong, Guangxi, Fujian, and Taiwan, as well as on Hainan Island.

### 3.2 Key environmental variables

In our analysis using the MaxEnt model, 17 environmental factors were considered to predict the habitat suitability of *P. santalinus*. Among these, the four most influential variables, which together accounted for 72.8% of the total contribution rate, were annual precipitation (bio12, contributing 26.1%), annual mean temperature (bio1, 16.8%), temperature seasonality (bio4, 16.1%), and mean diurnal range (bio2, 13.8%), as detailed in Table 2. Notably, temperature seasonality (bio4) emerged as the factor with the highest permutation importance, registering at 50.4%. Compared to climatic factors, soil and terrain exert a relatively minor influence on the distribution of *P. santalinus*, collectively accounting for 13.4% of the total contribution rate.

**TABLE 2:** The percentage contributions of each environmental variable in Maxent modeling.

Environmental factor	Percent contribution (%)	Permutation importance (%)
bio12	26.1	5.8
bio1	16.8	11
bio4	16.1	50.4
bio2	13.8	8.2
bio15	6	3.5
bio3	5.5	3.7
slope	4.8	1
t_oc	1.9	4.3
t_ph	1.8	1
aspect	1.8	0.6
t_gravel	1.7	1.5
bio14	1.6	2.8
alt	0.9	2.9
bio8	0.7	2.6
t_clay	0.3	0.2
t_sand	0.2	0.4
t_ref_bulk_density	‐	‐

**FIGURE 4:**
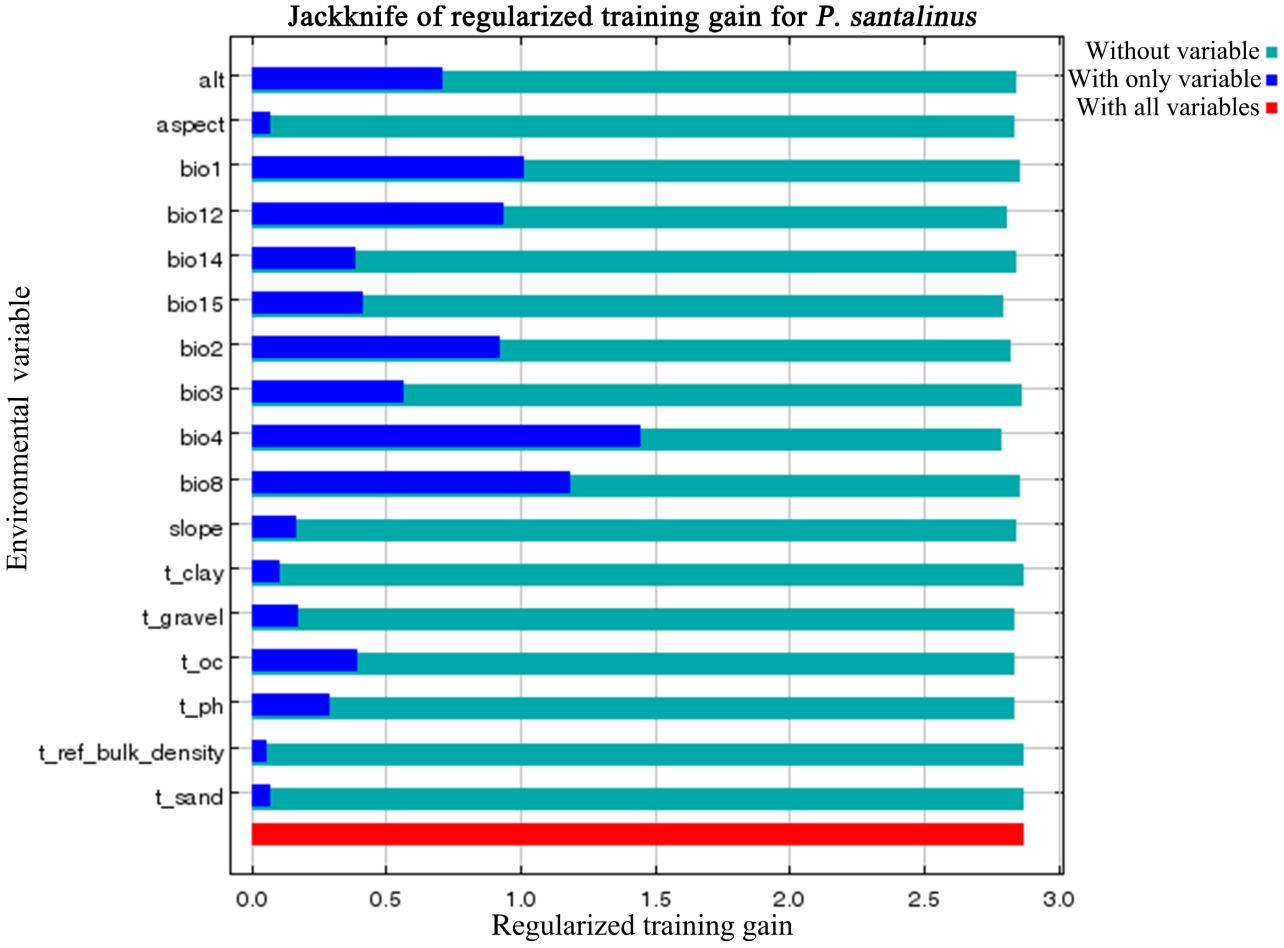
Jackknife test of variable importance for *Pterocarpus santalinus* in the MaxEnt modeling.

**FIGURE 5:**
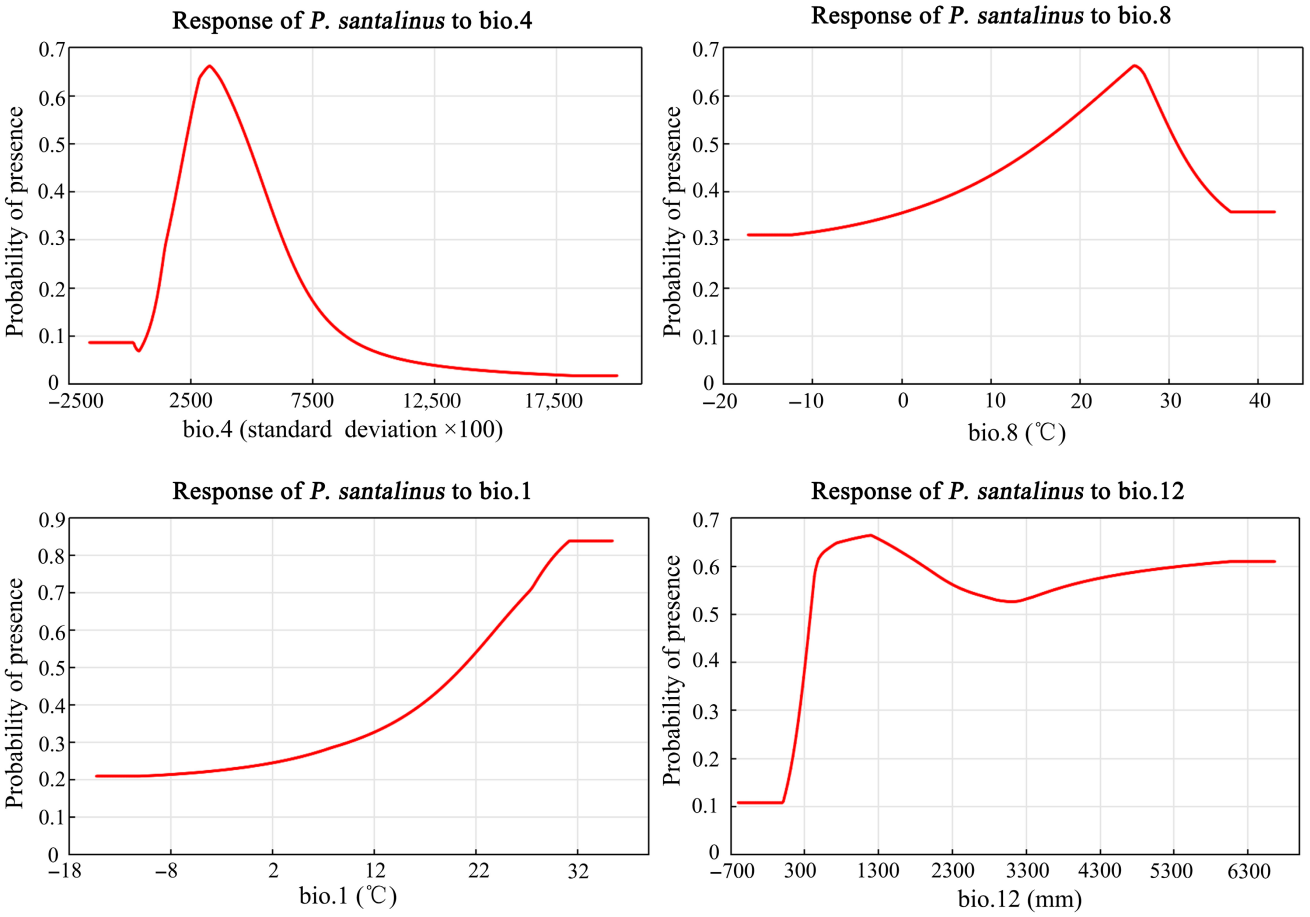
Response curves for the key environmental variables in the MaxEnt model for *Pterocarpus santalinus*.

The jackknife test results further reinforced the significance of these variables (Figure 4). When each variable was used in isolation, temperature seasonality (bio4) yielded the highest model gain, indicating its critical role in defining suitable habitats for *P. santalinus*. This was followed by the mean temperature of the wettest quarter (bio8), annual mean temperature (bio1), and annual precipitation (bio12). The response curves for these environmental variables (Figure 5) revealed the optimal conditions for *P. santalinus* growth: temperature seasonality (bio4) ranging from 2283.6 to 4882.3 (standard deviation × 100), mean temperature of the wettest quarter (bio8) between 15.6 and 30.8°C, annual mean temperature (bio1) from 20.7 to 35.4°C, and annual precipitation (bio12) spanning 388–6663 mm. These findings indicate that *P. santalinus* thrives in warm and humid environments. Furthermore, the results also showed that the impact of terrain and soil factors on the distribution of *P. santalinus* is relatively minor, whereas an increase in slope (0–90°) tends to favor the growth of *P. santalinus* (Figure S2).

### 3.3 Potential habitat change for *P. santalinus* in the future

**FIGURE 6:**
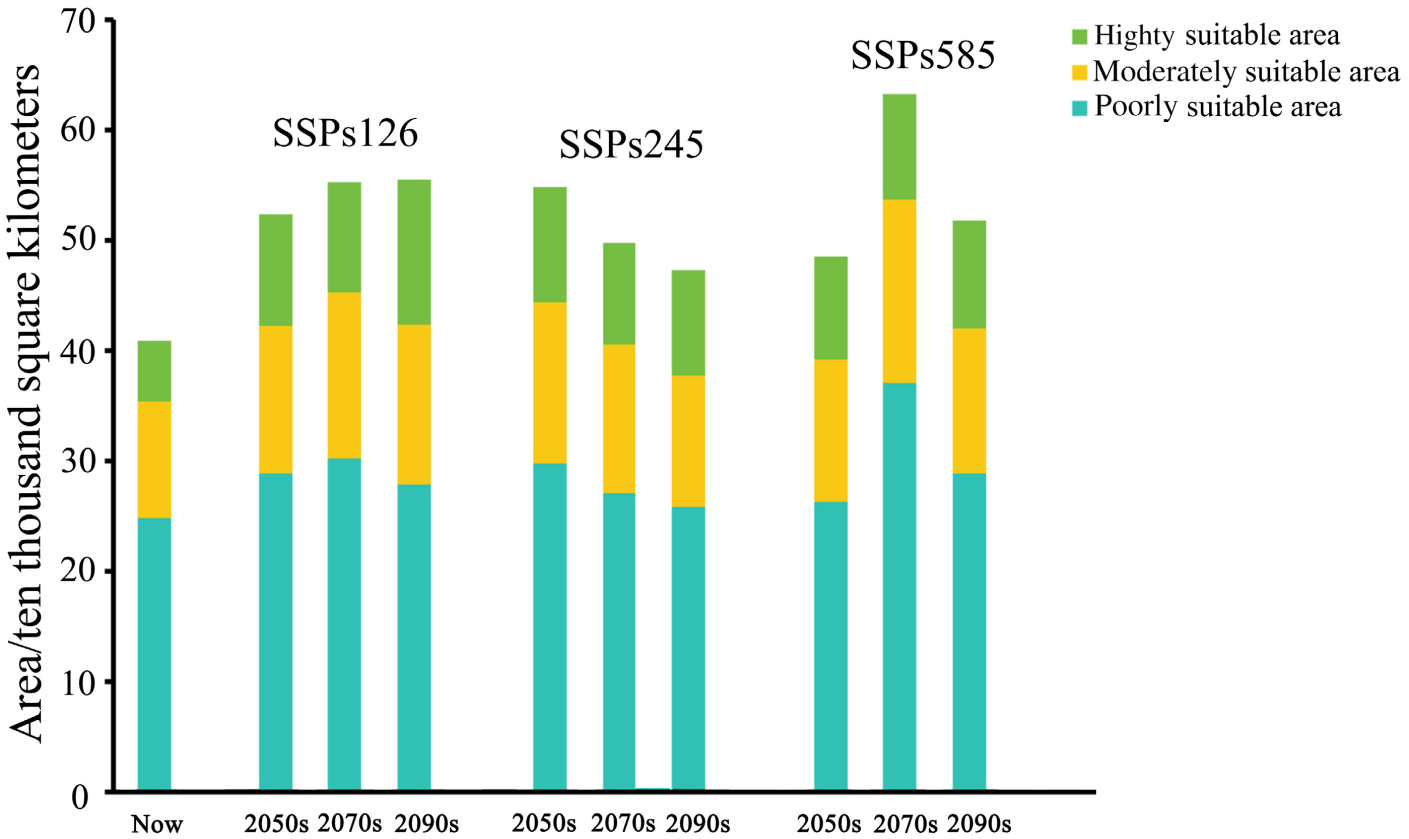
Suitable planting areas for *Pterocarpus santalinus* under current and future climatic conditions.

To assess the potential effects of climate change on *P. santalinus* habitats in China, we simulated habitat suitability changes using three future climate scenarios: SSPs126, SSPs245, and SSPs585. Our findings, illustrated in Figure 6, indicate an overall increase in the habitat area for *P. santalinus* across all scenarios. The expansion of moderately and highly suitable areas for *P. santalinus* is most pronounced under the SSPs126 scenario, with growth rates of 35.7% and 98.02%, respectively, surpassing those observed under the SSPs245 (26.22%, 74.63%) and SSPs585 scenarios (35.5%, 70.31%). This trend suggests that the intensity of climate change impacts the distribution of suitable habitats for *P. santalinus*, with milder climate change scenarios (like SSPs126) resulting in more favorable conditions for the species.

### 3.4 Expansion and shrinkage of the potential habitat in the future

**FIGURE 7:**
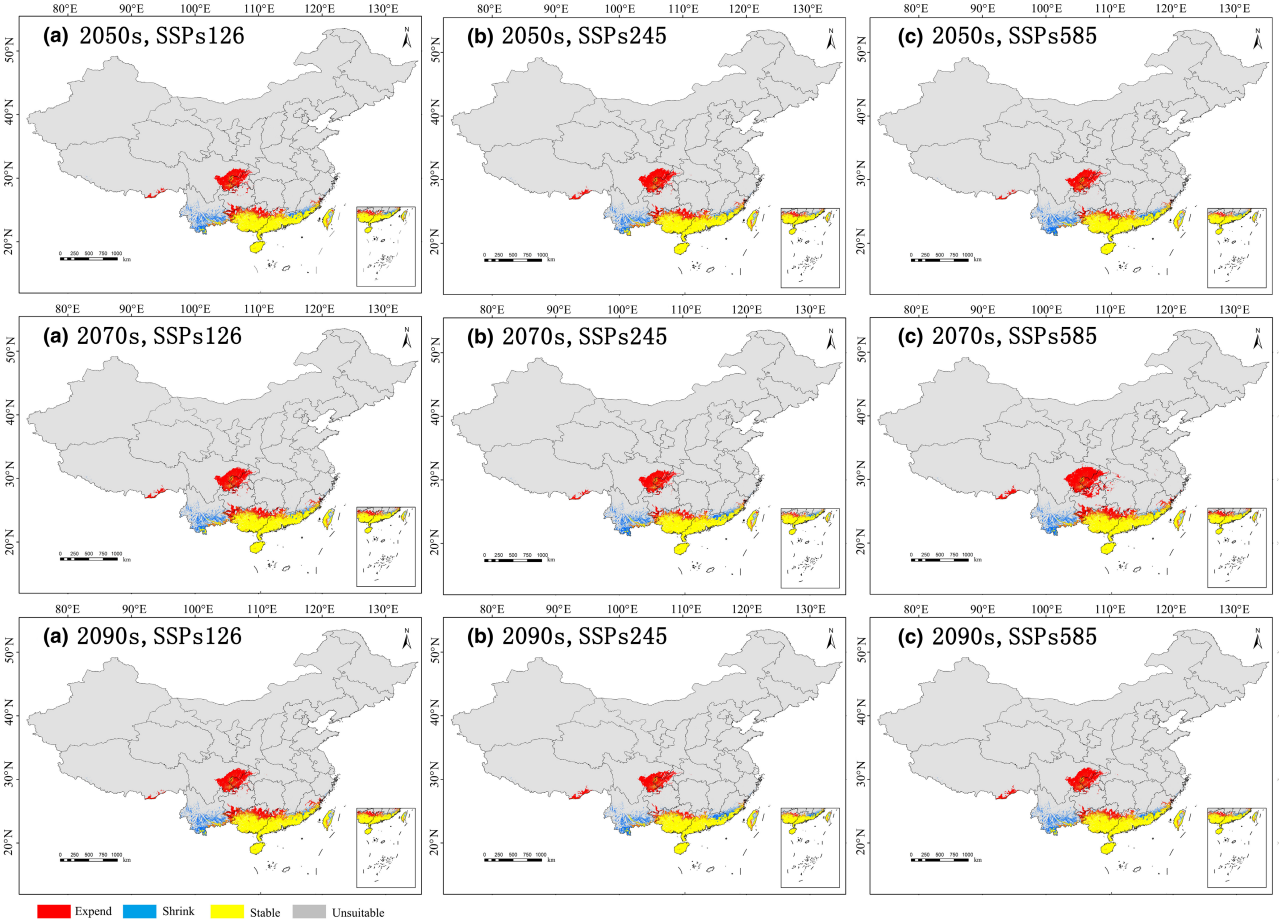
Changes in the distribution of suitable habitat for *Pterocarpus santalinus* under future climate conditions. (a) SSPs126 (2050s, 2070s,2090s), (b) SSPs245 (2050s, 2070s, 2090s), (c) SSPs585 (2050s, 2070s, 2090s).

To predict the future changes in suitable habitats for *P. santalinus*, we overlaid current data with future projections in ArcGIS, focusing on stable, shrinking, and expanding regions (Figure 7). The analysis revealed that under the three different emission scenarios (SSPs126, SSPs245, and SSPs585), the future distribution patterns of *P. santalinus* habitats share similarities. The stable areas are predominantly located in Hainan Island, in the central and in the southern parts of Guangdong and Guangxi, and the coastal areas of Fujian and Taiwan. In contrast, the shrinking habitats are primarily in central‐southern Yunnan, southern Fujian, northeast Guangdong, and central Taiwan. The expanding habitats are expected at the junction of Sichuan and Chongqing, in a northern Guangdong, in a northern Guangxi, and in a small part of southeast Xizang.

Under the SSPs126 scenario (Figure 7a), the suitable habitats (stable and expanding) for *P. santalinus* are projected to expand by 1.04% CA (current area) per 20 years. In contrast, under the SSPs245 scenario, the distribution of this species is anticipated to contract by 2.32% CA per 20 years, following a significant expansion by the 2050s. As for the SSP585 scenario, the distribution of *P. santalinus* is expected to rise by 3% CA from the 2050s to the 2070s, followed by a decrease of 1% CA by the year 2090s. The aforementioned results indicated that the dramatic climate changes seem to render the expansion of *P. santalinus* distribution areas unstable.

### 3.5 Trajectory changes of suitable habitat centroid of *P. santalinus* in the future

**FIGURE 8:**
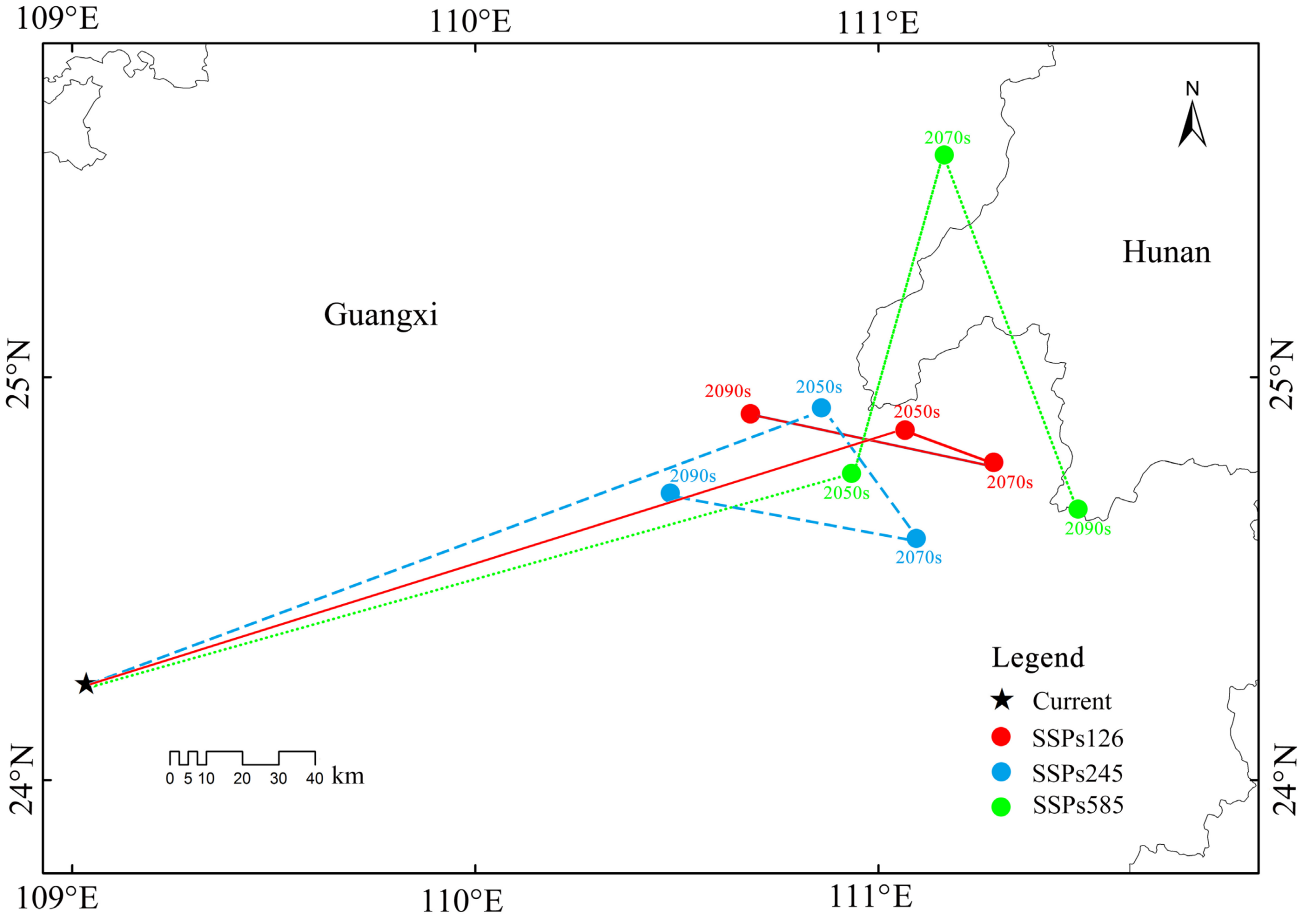
Trajectory changes of the centroid in the future for *Pterocarpus santalinus*.

The trajectory changes of the suitable habitat centroid of *P. santalinus* are depicted in Figure 8. Currently, the centroid of suitable habitats for *P. santalinus* is positioned at 109°1′ E, 24°13′ N. Under the SSPs126 scenario, there is a notable shift in the centroids over the decades: for the 2050s, it moves to 111°3′ E, 24°52′ N; for the 2070s, to 111°17′ E, 24°47′ N; and for the 2090s, it shifts to 110°40′ E, 24°54′ N. This trajectory indicates an initial northeastward movement, followed by a southeast turn, and ultimately a northwestward shift. A similar pattern is observed under the SSPs245 scenario, with the centroids located at 110°51′ E, 24°55′ N (2050s), 111°5′ E, 24°36′ N (2070s), and 110°29′ E, 24°43′ N (2090s), showing a northeastward movement initially, then southeast, and finally northwest. In contrast, under the SSPs585 scenario, the centroids are located at 110°56′ E, 24°45′ N (2050s), 111°10′ E, 25°33′ N (2070s), and 111°29′ E, 24°40′ N (2090s), indicating a consistent northeastward shift initially, followed by a southeast turn. Although the centroid of the *P. santalinus* distribution is projected to migrate in various directions under different future scenarios, a common trend is the expansion toward higher latitudes.

## 4 DISCUSSION

### 4.1 Predictive performance of MaxEnt model

While numerous climate modeling tools are continuously developed, MaxEnt remains a leading software for predicting the current and future geographic distribution of species (Iverson & Mckenzie, 2013). Its widespread application is evident in studies predicting suitable planting areas for various species, including *Pogostemon cablin* (Zeng et al., 2021), *Arabica coffee* (Zhang et al., 2021), and *Citrus medica* (Xia et al., 2023). In this study, we utilized MaxEnt to delineate the potential geographic distribution of *P. santalinus* under current and future climate scenarios. Our findings provide a scientific basis for the strategic planning and efficient cultivation of *P. santalinus* in China.

The response curve of the MaxEnt model has consistently been a focal point of analysis for predicting the potential distribution of plant species, offering an abundance of reference information for understanding the environmental requirements of plants (De Cauwer et al., 2014). The response curves indicated that the minimum temperature suitable for the growth of *P. santalinus* must exceed 15.6°C, and the annual precipitation should be above 388 mm. This suggested that *P. santalinus* has the capacity to thrive in high temperatures and humidity, while also demonstrating a certain level of drought resistance. For example, the seedling survival rate of *P. santalinus* is over 90% on both moist and arid soils in Kerala, India (Chandrashekara et al., 2001). By integrating bio4, bio8, and bio1, we deduced further that this species is more sensitive to rapid temperature fluctuations, which may impact its survival and growth, particularly under the unpredictable of climate change (Krasensky & Jonak, 2012). Relevant studies showed that the narrow isothermal range restricted the distribution range of *P. santalinus* in the Eastern Ghats, India (Babar et al., 2012). Furthermore, we observed that the ecological suitability of *P. santalinus* increased with steeper slopes. This suggested that the species exhibited a pronounced preference toward soils with enhanced drainage capabilities, which was similar to the environmental requirements of *P. angolensis* (De Cauwer et al., 2014). Compared to climatic variables, the impact of soil on the distribution of *P. santalinus* is relatively minor. As a leguminous plant, *P. santalinus* can form root nodules that accelerate the accumulation of nutrients in the soil and can grow normally in degraded lands (Chandrashekara et al., 2001; Rajasekhar et al., 2002), indicating that rosewood does not have strict soil requirements. Therefore, when selecting sites for the cultivation of *P. santalinus*, priority should be given to regions that offer a combination of higher ambient temperatures and adequate precipitation, coupled with well‐drained soils and minimal temperature fluctuations.

As a tropical species, the native habitat of *P. santalinus* features a warm and humid climate (Ankalaiah, 2022), analogous to the climatic conditions of the southern regions of China. Our predictive modeling suggested that *P. santalinus* was amenable to cultivation across select provinces within China, including Guangdong, Guangxi, Fujian, Yunnan, Hainan, Taiwan, and parts of Sichuan. Currently, five of the aforementioned provinces have documented the cultivation of *P. santalinus* (Xu et al., 2016). The consistency of our findings with the known climate conditions and distribution areas of *P. santalinus* (Ankalaiah, 2022; Xu et al., 2016) attests to the accuracy of the potential suitable habitats predicted by MaxEnt.

### 4.2 Changes in potential distribution and centroid of *P. santalinus* in the future

Due to the differential responses of various species to future climate change, some may potentially experience an expansion of their distributional ranges under the projected climatic conditions, whereas others could face a reduction in their habitats, consequently encountering significant survival challenges (Jinga & Ashley, 2019). Under future climate scenarios, we observe a notable northeastward migration trend in the suitable habitat for *P. santalinus*. This shift suggests an expansion in the areas conducive to the growth of *P. santalinus*, particularly evident under the SSPs585 climate scenario in the 2070s. The Intergovernmental Panel on Climate Change (IPCC) forecasts that global warming, predominantly driven by human activities, will persist (Parry et al., 2007). Supporting this, Jiang and Fu (2012) project an average temperature increase of 2.7–2.9°C in China, with relatively smaller temperature variations along the southeastern coast under various greenhouse gas emission scenarios. Additionally, average precipitation is expected to rise by 3.4%–4.4%, with variations across different regions of China.

These climatic changes will likely prompt the migration of some tropical and subtropical tree species to higher altitudes and latitudes, as observed in species like *Sapindus mukorossi* (Li et al., 2022), *Cyclobalanopsis glauca* (Zhang et al., 2022), and *Pistacia chinensis* (Xu et al., 2023). Specifically for *P. santalinus*, the expanding habitat under future climate conditions is predicted to be concentrated around the junction of Sichuan and Chongqing, northern Guangdong, northern Guangxi, and parts of southeast Xizang. Hence, future climatic alterations may engender positive implications for the cultivation zones of *P. santalinus* within China, with analogous reports having been found in *P. erinaceus* and *P. marsupium* (Adjonou et al., 2020; Khanal et al., 2022).

It is noteworthy that the suitability of the new niches for the cultivation of *P. santalinus* is not absolute. It remains subject to constraints imposed by other environmental factors, such as competition among flora, wildfires, human activities, and diseases and pests (Gbenyedji et al., 2016; Ivory & Russell, 2016; Trisurat et al., 2016). For example, *P. santalinus* trees are frequently subjected to herbivory by certain insects, such as seed borer or leaf‐eating caterpillars (Umalatha & Anuradha, 2019). Second, global warming is anticipated to increase the frequency and intensity of extreme weather events, leading to greater temperature fluctuations (Yeh et al., 2018). In some regions of China, such extreme events may result in abnormal warmth or increased precipitation (Garfinkel et al., 2013; Weng et al., 2007), which may potentially create more suitable habitats for *P. santalinus*. However, the accompanying shifts in seasonal temperatures might counteract this trend of habitat expansion. Our study observed an increase in the suitable habitat of *P. santalinus* under the SSPs126 climate scenario, whereas under the SSPs245 and SSPs585 scenarios, the habitat initially expanded but then contracted. Thirdly, the changes in land use and cover significantly affect *P. santalinus*, particularly as its potential distribution areas identified through ecological niche modeling mainly fall in regions under high anthropogenic pressure. Anthropogenic logging and habitat destruction are considered to be among the primary factors contributing to the endangerment of *P. santalinus* (Arunkumar & Joshi, 2014).

Our research established the suitable planting areas for *P. santalinus* in China. However, as an introduced species, the establishment of *P. santalinus*'s distribution area in China still depends on the species' dispersal and reproductive capabilities. This species has been noted for its low natural regeneration, possibly due to slow growth and recruitment challenges. *P. santalinus* predominantly produces fruit through cross‐pollination, with bees serving as the primary pollinators. The low fruiting rate of around 6% highlights the sensitivity of this species to effective pollination dynamics (Rao & Raju, 2002). Furthermore, the bottleneck in maturation from juvenile to adult trees raises concerns for future populations (Ankalaiah et al., 2017). Considering the aforementioned factors, devising a rational planting strategy can help mitigate potential economic losses and achieve more efficient cultivation of *P. santalinus* in the face of ongoing climate change.

### 4.3 Limitations of the study

By incorporating climate, soil, and terrain factors, our study contributes valuable insights into how *P. santalinus* responds to future climate change, which is essential for optimizing its cultivation and planting strategies. However, it is undeniable that there are inherent limitations in predicting a species' habitat through modeling.

1. Data Quality and Availability: One fundamental limitation is the reliance on the quality and availability of input data. MaxEnt predictions are as good as the data fed into them, which includes occurrence records and environmental layers. Incomplete or biased occurrence data can lead to skewed predictions, and this limitation should be acknowledged.
2. Model Assumptions: The MaxEnt model, like all species distribution models, makes several assumptions. It assumes niche equilibrium, meaning it presumes that species distributions are in equilibrium with the current climate. This may not be true, especially for species experiencing rapid environmental changes or those with dispersal limitations.
3. Static Climate Variables: The environmental variables used in MaxEnt are typically static, representing a snapshot in time. This does not account for the dynamic nature of both the climate and species' responses, which may involve lag effects or evolutionary adaptations. Such dynamics are difficult to incorporate into the model but critical for understanding future distributions.
4. Exclusion of Biotic Interactions: MaxEnt primarily considers abiotic factors (like climate and terrain) and generally overlooks biotic interactions such as competition, predation, and symbiosis, which can significantly affect species distribution and abundance.
5. Spatial and Temporal Scale: The predictions made by MaxEnt are sensitive to the spatial and temporal scales of the data and analysis. Results might differ if the model is applied to different geographic extents or temporal periods, which may not have been fully explored in this study.
6. Impact of Extreme Events: While climate averages are crucial for distribution modeling, extreme climatic events (which are likely to increase under climate change scenarios) can also have significant impacts on species survival and distribution. MaxEnt does not typically account for these extremes, potentially overlooking critical thresholds for species survival.
7. Generalization of Results: The study's findings are based on specific climate scenarios and might not generalize to other scenarios or geographic regions. This limitation should be addressed, especially when considering the application of the results to conservation strategies and policymaking.
8. Predictive Uncertainty: There is inherent uncertainty in any predictive modeling. Quantifying the uncertainty in the predictions of *P. santalinus* distribution under future climate scenarios can help in better understanding the confidence in the projections and is often an overlooked aspect of species distribution modeling.

### 4.4 Future prospect

To enhance the accuracy of future predictions, it will be beneficial to integrate both environmental and biological factors into our models. This comprehensive approach can offer a more holistic understanding of the suitable habitat for *P. santalinus*, thus aiding in the development of more effective strategies for its management and cultivation in the face of climate change.

## 5 CONCLUSIONS

The study on *P. santalinus* in China, utilizing the MaxEnt model and considering various future climate scenarios, concludes that the species' suitable habitats are likely to expand, particularly under the SSPs126, SSPs245, and SSPs585 climate scenarios, with a notable northeastward migration of the distribution center in the 2050s, 2070s, and 2090s. This expansion and migration are attributed to the species' sensitivity to climatic changes, especially temperature fluctuations. Key environmental factors such as annual precipitation, mean temperature, and temperature seasonality have been identified as critical in influencing the distribution of *P. santalinus*. These findings provide a crucial scientific basis for the strategic planning and efficient cultivation of *P. santalinus* in China, highlighting the importance of considering both environmental and biological factors in future research to address the challenges of climate change, pest outbreaks, and other biological stressors. This comprehensive understanding is vital for developing adaptive strategies for the cultivation and conservation of this economically significant species in the face of ongoing climatic shifts.

## AUTHOR CONTRIBUTIONS

**Xiao‐Feng Zhang:** Project administration (equal); software (equal); writing – original draft (equal); writing – review and editing (equal). **Mir Muhammad Nizamani:** Methodology (equal); software (equal); supervision (equal); validation (equal); writing – review and editing (equal). **Chao Jiang:** Conceptualization (equal); formal analysis (equal); methodology (equal); validation (equal). **Fa‐Zhi Fang:** Data curation (equal); investigation (equal). **Kun‐Kun Zhao:** Supervision (equal); validation (equal); visualization (equal); writing – original draft (equal); writing – review and editing (equal).

## FUNDING INFORMATION

This study was supported by the Hainan Academy of Forestry (Hainan Academy of Mangrove), Grant/Award Number: KYYSLK2023‐018.

## CONFLICT OF INTEREST STATEMENT

The authors declare no conflicts of interest.

## Supporting information


Figure S1

Figure S2

## Data Availability

The original datasets have been uploaded to an open data repository, Dryad (DOI: 10.5061/dryad.0p2ngf274).
