# Supplementary figures and images for: Potential planting regions of Pterocarpus santalinus (Fabaceae) under current and future climate in China based on MaxEnt modeling
Source: Ecol Evol. 2024 May 30;14(6):e11409. doi: 10.1002/ece3.11409 (PMC11139971; doi:10.1002/ece3.11409)

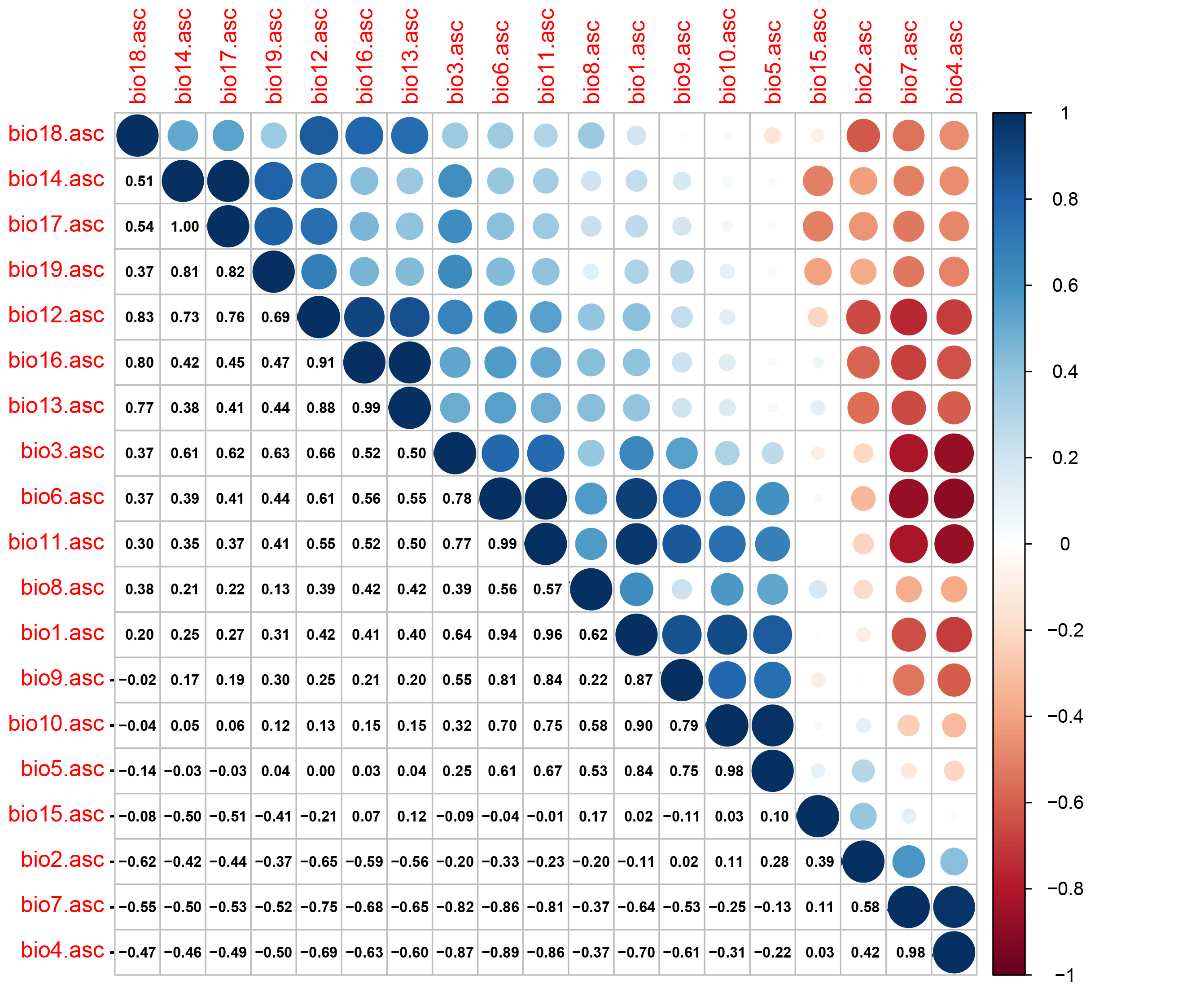

Supplement: Supplementary file 1 — Figure S1 [file ECE3-14-e11409-s001.jpg]
